# Integrated Histopathologic and Targeted Genomic Characterization of Gastric Adenocarcinomas with Yolk Sac Tumor Differentiation

**DOI:** 10.3390/ijms27135786

**Published:** 2026-06-26

**Authors:** Annabella Di Mauro, Rosalia Anna Rega, Rosalinda Sorrentino, Anna Falanga, Maddalena Leongito, Vittorio Albino, Andrea Belli, Imma D’Arbitrio, Saverio Simonelli, Rossella De Cecio, Salvatore Tafuto, Guglielmo Nasti, Alessandro Ottaiano, Fabiana Tatangelo

**Affiliations:** 1 Pathology Unit, Istituto Nazionale Tumori, IRCCS-Fondazione “G. Pascale”, Via Mariano Semmola, 80131 Napoli, Italy; rosalia.rega@istitutotumori.na.it (R.A.R.); imma.darbitrio@istitutotumori.na.it (I.D.); saverio.simonelli@istitutotumori.na.it (S.S.); r.decio@istitutotumori.na.it (R.D.C.); fabiana.tatangelo@istitutotumori.na.it (F.T.); 2 Department of Pharmacy, University of Salerno, 84084 Fisciano, Italy; rosalinda.sorrentino@unisa.it (R.S.); anna.falanga@unisa.it (A.F.); 3 Department of Gastro-Hepato-Pancreato-Biliary Surgery, Istituto Nazionale Tumori, IRCCS-Fondazione “G. Pascale”, Via Mariano Semmola, 80131 Napoli, Italy; maddalena.leongito@istitutotumori.na.it (M.L.); v.albino@istitutotumori.na.it (V.A.); a.belli@istitutotumori.na.it (A.B.); 4 Sarcomas and Rare Tumors Unit, Istituto Nazionale Tumori, IRCCS-Fondazione “G. Pascale”, 80131 Naples, Italy; s.tafuto@istitutotumori.na.it; 5 Division of Innovative Therapies for Abdominal Metastases, Istituto Nazionale Tumori, IRCCS-Fondazione “G. Pascale”, 80131 Naples, Italy; g.nasti@istitutotumori.na.it (G.N.); a.ottaiano@istitutotumori.na.it (A.O.)

**Keywords:** gastric tumors, yolk sac differentiation, next-generation sequencing, PI3K-AKT pathway, rare tumors

## Abstract

Gastric adenocarcinomas with yolk sac tumor (YST) differentiation represent an exceptionally rare and poorly understood phenotype, characterized by the emergence of extraembryonic features within an epithelial malignancy. Their histogenesis remains debated, with increasing evidence supporting somatic lineage plasticity rather than germ cell origin. Here, we performed an integrated histopathologic and genomic characterization of three gastric adenocarcinomas with YST differentiation surgically treated at a tertiary cancer center. Histologically, all tumors showed a predominant adenocarcinoma component associated with variable YST differentiation, displaying reticular/microcystic and papillary patterns and expression of oncofetal markers, including alpha-fetoprotein (AFP) and Glypican-3. Targeted next-generation sequencing using a 523-gene panel revealed microsatellite-stable profiles with intermediate tumor mutational burden and substantial intertumoral heterogeneity. Despite gene-level variability, the detected alterations involved signaling pathways commonly implicated in epithelial tumorigenesis, including PI3K-AKT and RTK/RAS-MAPK signaling. Several recurrent alterations were identified across cases, including CCND3 variants and MDM2 copy number gains; however, their biological significance requires validation in larger cohorts. Functional enrichment analysis identified alterations involving developmental and proliferative signaling programs. Overall, these findings suggest that YST differentiation may represent a phenotypic manifestation of epithelial tumor plasticity arising within gastric adenocarcinoma and is associated with epithelial-related oncogenic programs, although broader genomic and comparative studies are required to clarify its histogenesis. This study provides preliminary molecular and histopathologic insights into this rare entity and supports the integration of molecular profiling into its diagnostic and translational management.

## 1. Introduction

Yolk sac tumor (YST) is a highly aggressive malignant neoplasm within the germ cell tumor spectrum, characterized by primitive extraembryonic differentiation and marked biological heterogeneity [1,2]. YSTs most commonly arise in the gonads; however, extragonadal presentations along midline anatomical structures have been described, reflecting aberrant developmental pathways during embryogenesis [2]. In contrast, primary involvement of the stomach is exceedingly rare, with only a limited number of cases reported in the literature.

A distinctive feature of gastric tumors with yolk sac differentiation is their frequent association with conventional gastric adenocarcinoma. Rather than representing pure germ cell neoplasms, these tumors typically exhibit a composite morphology, in which a conventional adenocarcinoma component coexists with areas of yolk sac differentiation [3,4,5,6]. This clinicopathological setting has fueled a longstanding debate regarding histogenesis. While early hypotheses favored aberrant germ cell migration, increasing morphological and molecular evidence supports an alternative model of somatic transformation. In this context, adenocarcinoma is thought to undergo aberrant retrodifferentiation into yolk sac-like elements [6,7]. Molecular clonality studies demonstrating shared genetic alterations between glandular and YST components further support the concept of lineage plasticity rather than the collision of independent tumors [8].

From a diagnostic standpoint, the identification of yolk sac differentiation relies on the recognition of characteristic architectural patterns, including reticular/microcystic growth, papillary or glandular structures, and endodermal sinus-like formations such as Schiller–Duval bodies. However, given the marked morphological heterogeneity and potential overlap with poorly differentiated adenocarcinoma, immunophenotypic confirmation is essential [9,10]. In this setting, markers of germ cell and oncofetal differentiation play a central role. Expression of SALL4, Glypican-3, and alpha-fetoprotein (AFP) is commonly observed and remains diagnostically informative [11,12,13], whereas OCT3/4 and PLAP are typically associated with other germ cell tumor subtypes and may aid in the differential diagnosis. Notably, YSTs may exhibit considerable phenotypic heterogeneity, including aberrant expression of divergent endodermal lineage markers, reflecting their primitive and pluripotent nature [14,15].

More broadly, developmental reprogramming is increasingly recognized as a hallmark of tumor lineage plasticity, a process implicated in tumor progression, therapeutic resistance, and phenotypic diversification [16,17]. Within this framework, the concept of oncofetal reprogramming has emerged, describing the reactivation of embryonic transcriptional programs that promote stemness, immune evasion, and cellular adaptability during tumor evolution [18,19]. Gastric tumors with yolk sac differentiation, characterized by the expression of oncofetal proteins and primitive morphology, may represent an extreme manifestation of this phenomenon [20].

Despite advances in large-scale cancer sequencing efforts, the genomic landscape of gastric adenocarcinomas with yolk sac differentiation remains poorly defined [21]. This gap is clinically relevant, as it limits the development of biologically informed diagnostic and therapeutic strategies for a tumor subtype often associated with aggressive clinical behavior. Moreover, no standardized treatment approaches are currently established [22,23]. In the era of precision oncology, comprehensive genomic profiling has demonstrated increasing utility in rare and refractory malignancies, enabling the identification of actionable alterations beyond conventional histology-based paradigms [24,25,26].

However, it remains unclear whether gastric tumors with yolk sac differentiation recapitulate the canonical molecular features of germ cell tumors or instead reflect epithelial-driven oncogenic programs associated with lineage plasticity.

In this study, we aimed to provide an integrated histopathological and molecular characterization of gastric adenocarcinomas with yolk sac tumor differentiation using targeted next-generation sequencing. We hypothesized that yolk sac differentiation may represent a phenotypic manifestation of epithelial tumor plasticity associated with epithelial-related oncogenic programs. By combining morphologic, immunophenotypic, and genomic analyses, we sought to provide an integrated histopathologic and molecular characterization of this rare tumor phenotype.

## 2. Results

### 2.1. Clinicopathologic Features

Three cases of gastric adenocarcinoma with yolk sac tumor (YST) differentiation were identified and surgically treated at the National Cancer Institute of Naples (IRCCS Fondazione G. Pascale). All patients were male (age range: 55–75 years) and presented with ulcerated lesions involving the gastric antrum or fundus/stump. Detailed clinicopathologic characteristics, including pathologic staging and clinical outcomes, are summarized in Table 1. All cases were treatment-naïve at the time of molecular analysis.

**Figure 1:**
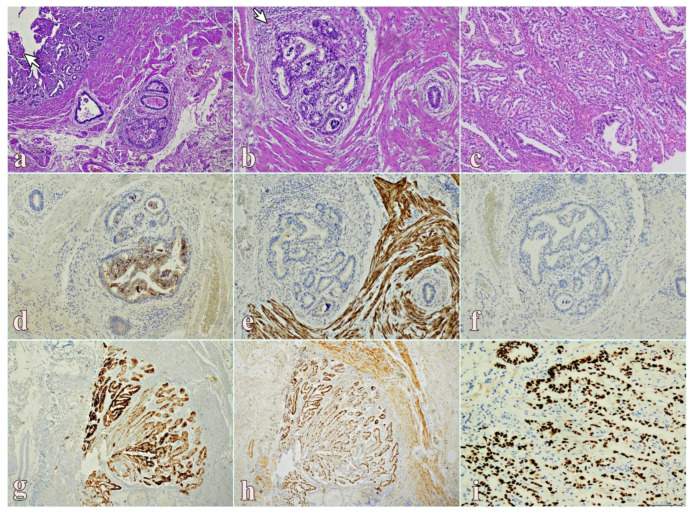
Morphologic and immunophenotypic features of primary gastric tumor with yolk sac differentiation. (**a**) Low-power view showing the composite nature of the neoplasm, with conventional tubular adenocarcinoma admixed with a primitive reticular/microcystic component consistent with yolk sac tumor (YST) differentiation (H&E, ×4). (**b**) Higher magnification of the yolk sac tumor component (H&E, ×20). (**a**,**b**) Arrows indicate areas containing Schiller–Duval body-like structures. (**c**) Additional areas show complex glandular architecture within desmoplastic stroma (H&E, ×10). (**d**) Cytoplasmic positivity for alpha-fetoprotein (AFP) in the yolk sac tumor component. (**e**) Absence of nuclear expression of OCT3/4. (**f**) Negative staining for placental alkaline phosphatase (PLAP). (**g**) Cytoplasmic positivity for Glypican-3 in the yolk sac tumor component (×20). (**h**,**i**) Diffuse strong nuclear expression of SALL4 in the yolk sac tumor component, supporting primitive germ cell differentiation (×4, ×20).

Histologically, all tumors exhibited a composite morphology characterized by a predominant conventional adenocarcinoma component associated with variable areas of yolk sac differentiation. The YST component accounted for an estimated proportion ranging from approximately 20% to 50% of the tumor volume, based on histologic evaluation. The yolk sac areas displayed a spectrum of architectural patterns, predominantly reticular/microcystic, with focal papillary or micropapillary growth (Figure 1a). Neoplastic cells showed marked cytologic atypia, including hyperchromatic nuclei, high nuclear-to-cytoplasmic ratios, and prominent nucleoli, with focal intra- and extracellular hyaline globules. At higher magnification, characteristic Schiller–Duval body-like structures were identified within the YST component (Figure 1b). Additional areas demonstrated complex glandular architecture within a desmoplastic stroma, consistent with the adenocarcinoma component (Figure 1c).

Immunohistochemically, the YST component showed strong cytoplasmic expression of alpha-fetoprotein (AFP) (Figure 1d) and Glypican-3 (Figure 1g), along with diffuse nuclear positivity for SALL4 (Figure 1h,i), supporting primitive extraembryonic differentiation. In contrast, OCT3/4 (Figure 1e) and placental alkaline phosphatase (PLAP) (Figure 1f) were negative, excluding alternative germ cell tumor subtypes.

The adenocarcinoma component lacked expression of germ cell markers and retained epithelial differentiation. Serum AFP levels were elevated in the available clinical records, supporting the diagnosis; however, complete serologic data were not available for all patients.

Pathologic staging classified two tumors as advanced (pT3) and one as an early-stage lesion (pT1b). All patients had regional lymph node metastases at diagnosis (pN1), and one case showed synchronous peritoneal dissemination. HER2 immunohistochemical expression was heterogeneous, ranging from score 0 to 2+ (Table 1). Clinical follow-up indicated an aggressive clinical course in advanced-stage cases, with two patients dying of disease at 11 months and 2 years, respectively, whereas the patient with pT1b disease remains alive and disease-free after 8 years of follow-up.

### 2.2. Genomic Landscape of Gastric Tumors with Yolk Sac Differentiation

**Figure 2:**
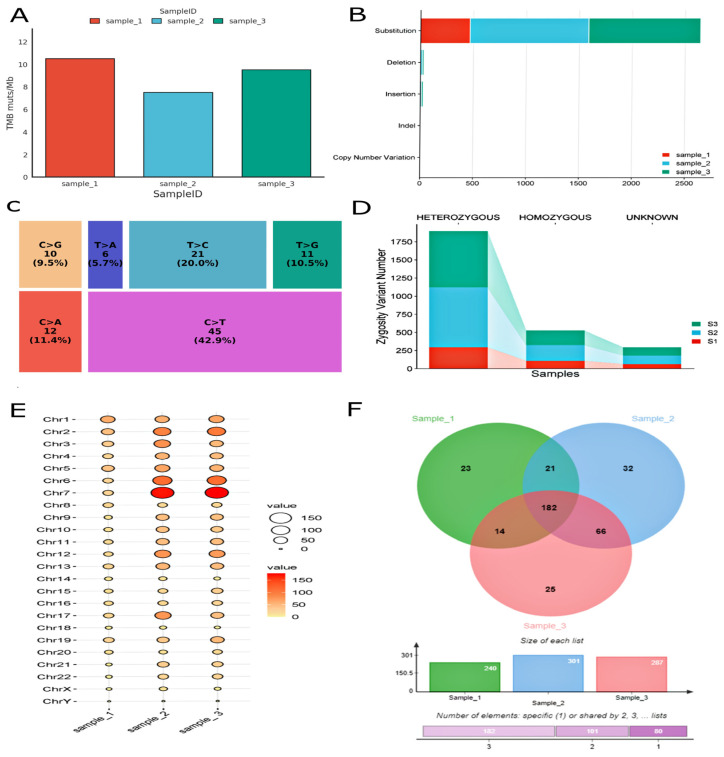
Integrated genomic landscape of primary gastric tumors with yolk sac differentiation. (**A**) Tumor mutational burden (TMB) across the three cases, indicating an intermediate mutational load. (**B**) Somatic variant classification, dominated by single-nucleotide variants (SNVs). (**C**) Single base substitution spectrum according to the COSMIC pyrimidine classification framework, showing predominance of C > T transitions. (**D**) Zygosity distribution demonstrating a predominance of heterozygous variants. (**E**) Genome-wide chromosomal distribution of somatic variants, with higher mutation density on chromosomes 7, 6, and 2. (**F**) Variant overlap across tumors, identifying a subset of shared alterations together with case-specific private mutations, highlighting intertumoral heterogeneity.

Targeted next-generation sequencing (NGS) was performed to characterize the somatic genomic landscape of gastric adenocarcinomas with yolk sac tumor (YST) differentiation. All cases were microsatellite-stable (MSS) and exhibited an intermediate tumor mutational burden (Figure 2A), showing molecular features more commonly reported in gastric adenocarcinoma than in canonical germ cell tumors. Across samples, the somatic variant spectrum was dominated by single-nucleotide variants (SNVs), with missense substitutions representing the most frequent alteration class (Figure 2B). Mutational spectrum analysis using the COSMIC pyrimidine classification framework demonstrated a predominance of C > T transitions (Figure 2C), consistent with age-related and clock-like mutational processes commonly observed in gastric adenocarcinoma.

Assessment of variant zygosity demonstrated a predominance of heterozygous alterations (Figure 2D), supporting a largely diploid genomic background with evidence of genomic heterogeneity. Somatic variants were broadly distributed across the genome, with higher mutation density observed on chromosomes 7, 6, and 2 (Figure 2E).

Comparative analysis of variant overlap identified a limited subset of alterations shared across all three tumors (n = 182); however, given the small cohort size and the targeted nature of the sequencing approach, these findings should be considered exploratory (Figure 2F). Overall, the tumors displayed substantial gene-level heterogeneity, with only partial overlap in the affected pathways across cases. Although several alterations involved signaling networks commonly implicated in epithelial tumorigenesis, these observations remain hypothesis-generating and require validation in larger cohorts. Collectively, the data support a heterogeneous molecular landscape and suggest that yolk sac differentiation in gastric neoplasms may be associated with epithelial-related oncogenic programs; however, these observations remain exploratory. Copy number profiling further revealed recurrent gains affecting key regulators of oncogenic signaling and cell-cycle control. *MDM2* amplification was detected in two tumors as the only recurrent copy number event, whereas two cases harbored ERBB2 amplification, subsequently confirmed by FISH analysis (estimated copy number = 61). Additional variable gains involved *CCND3*, *FGF19*, *BRCA2*, and *KRAS* (Supplementary Figure S1). The complete list of genomic alterations, including SNVs and copy number changes, is provided in Supplementary Table S1.

### 2.3. Functional Annotation of Genomic Alterations

To define the biological significance of the detected alterations, functional enrichment analyses were performed, exploring potentially affected signaling pathways underlying gastric adenocarcinomas with yolk sac tumor (YST) differentiation. Despite marked heterogeneity at the level of individual genes, pathway-level interrogation identified enrichment of signaling networks involved in cell proliferation, survival, and developmental reprogramming.

**Figure 3:**
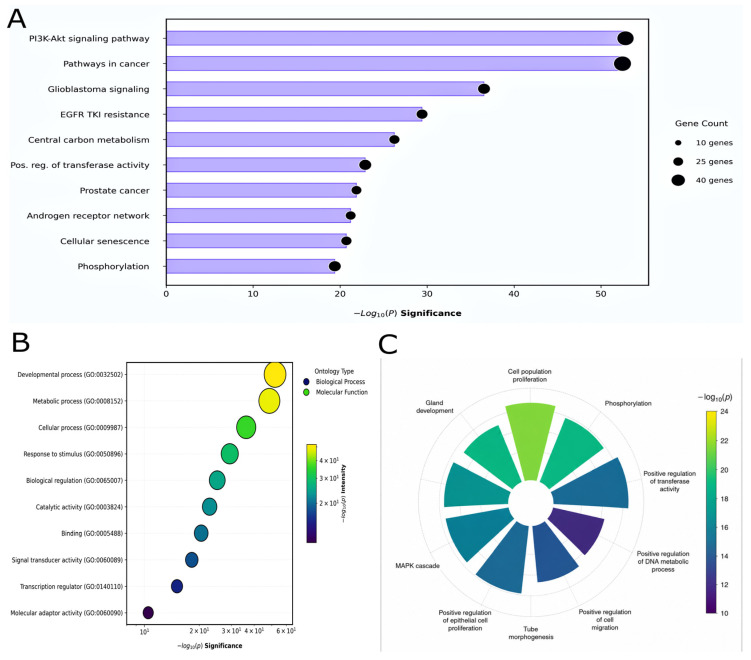
Multi-level functional enrichment analysis of genomic alterations. (**A**) Pathway enrichment results ranked by −log10(*p*) significance, with bubble size proportional to gene count; PI3K-AKT signaling was among the enriched pathways identified. (**B**) GO-Slim enrichment bubble plot summarizing overrepresented Biological Process and Molecular Function categories according to statistical significance and gene number (**C**) Circular bar plot highlighting selected enriched biological processes, with bar height and color intensity reflecting −log10(*p*) values.

Notably, PI3K–AKT signaling was among the pathways identified in the enrichment analysis (Figure 3A), driven by heterogeneous but functionally convergent alterations, including high-level *ERBB2* amplification and recurrent gains affecting receptor tyrosine kinases (RTKs) such as *KIT* and *PDGFRA*. These observations suggest partial overlap in biologically relevant signaling pathways despite underlying genomic variability.

Gene Ontology (GO) analysis further highlighted enrichment of biological processes related to developmental regulation, metabolic control, cellular response to external stimuli, and modulation of cell–cell communication (Figure 3B). At the molecular function level, altered genes were predominantly associated with binding and catalytic activities, with additional enrichment in signal transduction and transcriptional regulatory functions (Figure 3B and Supplementary Figure S2).

Collectively, these exploratory findings suggest that some genomic alterations may involve developmental and proliferative signaling pathways; however, these observations require validation in larger cohorts and broader genomic studies. Overall, these exploratory findings suggest partial overlap in biologically relevant signaling pathways; however, the limited cohort size and targeted sequencing approach preclude definitive conclusions regarding pathway dependency or histogenesis.

### 2.4. Clinically Relevant Genomic Alterations

To evaluate the translational relevance of the identified somatic alterations, genomic variants were annotated using two independent clinical interpretation platforms (OncoDNA OncoKDM and PierianDx Clinical Genomic Workspace). Potentially actionable alterations were identified across the cohort, with a mean of three trial- or therapy-matched alterations per tumor (range: 1–5), according to platform-specific evidence frameworks.

**Figure 4:**
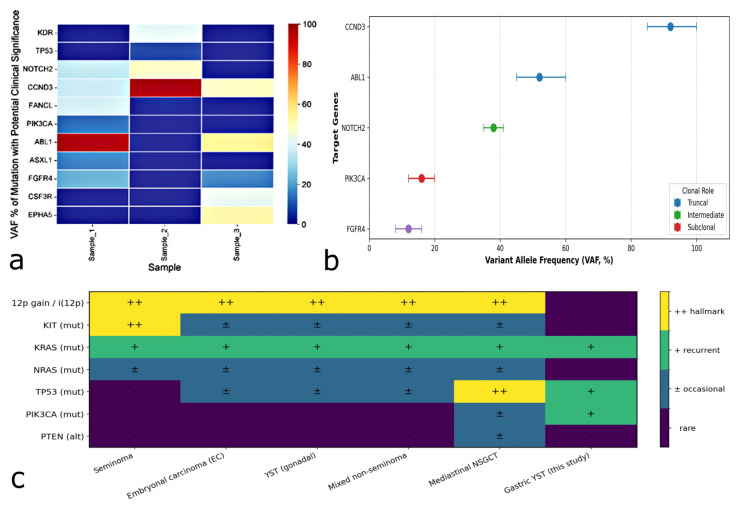
Distribution of selected clinically relevant variants according to variant allele frequency (VAF). (**a**) Heatmap of variant allele frequency (VAF, %) for selected alterations across the three tumors; color intensity reflects VAF; (**b**) Distribution of selected variants according to variant allele frequency (VAF); (**c**) Qualitative spectrum map comparing canonical genomic alterations in germ cell tumors with the profile observed in gastric tumors with YST differentiation. Symbols indicate alteration frequency (– rare, ± occasional, + recurrent, ++ hallmark).

Integrative analysis of mutational distribution and variant allele frequency (VAF) highlighted inter-case heterogeneity and variable variant allele frequencies. A high-resolution heatmap of clinically relevant variants (Figure 4a) illustrates the distribution of these alterations, highlighting both shared genomic events across the cohort and patient-specific alterations. Notably, a recurrent missense variant in *CCND3* (p.S259A) was identified in all three cases, with consistently high VAFs (range: 35.6–99.1%), suggesting that this alteration may represent a recurrent finding warranting further investigation in larger cohorts.

Variant allele frequency distribution analysis based on VAF distribution (Figure 4b) is compatible with intratumoral genomic heterogeneity. High-VAF alterations in *CCND3* and *ABL1* may represent alterations with relatively high VAFs, although definitive clonal inference cannot be established from targeted sequencing alone. In contrast, mutations in *PIK3CA* and *FGFR4* exhibited lower VAFs, indicative of lower VAF alterations potentially reflecting intratumoral heterogeneity. High-level ERBB2 amplification was identified in two tumors and subsequently confirmed by FISH. This alteration, together with recurrent *MDM2* gains, represents a potentially actionable genomic feature (Table 2). Although observed in a limited cohort, these alterations highlight candidate therapeutic vulnerabilities and suggest that targeted strategies, including HER2-directed therapies (e.g., trastuzumab deruxtecan, T-DXd) and MDM2 inhibitors, may be considered in selected cases within a precision oncology framework.

To contextualize these findings, we compared the molecular profile of gastric adenocarcinomas with yolk sac tumor (YST) differentiation to the canonical genomic landscape of gonadal and extragonadal germ cell tumors. A qualitative spectrum map (Figure 4c) indicates that these tumors only partially recapitulate the molecular hallmarks of classical germ cell malignancies, such as chromosome 12p gain or KIT-driven oncogenesis.

Instead, the alterations observed in our cohort converge on oncogenic pathways more typically associated with gastrointestinal carcinogenesis, including *ERBB2* amplification, *MDM2* gain, and PI3K pathway activation. These findings are compatible with epithelial-associated oncogenic programs, although broader comparative molecular studies are required to further clarify the relationship with canonical germ cell tumor biology, providing a rationale for prioritizing therapeutic strategies aligned with gastric adenocarcinoma-related dependencies.

### 2.5. Validation of Clinically Relevant Biomarkers and Copy Number Alterations

To validate the copy number alterations identified by targeted next-generation sequencing and further characterize clinically relevant biomarkers, fluorescence in situ hybridization (FISH) and additional immunohistochemical analyses were performed.

FISH confirmed high-level ERBB2 (HER2) amplification in two cases, characterized by increased gene copy number and focal signal clustering. These results are consistent with sequencing-based estimates and support the activation of HER2-driven oncogenic signaling. The remaining case was negative for ERBB2 amplification. MDM2 status was more heterogeneous. One tumor showed true MDM2 amplification, while another demonstrated chromosome 12 polysomy with increased MDM2 copy number, but no gene clustering or elevated MDM2/CEN12 ratio. This is consistent with low-level copy gain rather than focal amplification. Representative ERBB2, MDM2 and FGFR2 FISH findings are shown in Supplementary Figure S3.

Due to the therapeutic relevance of FGFR2 alterations in gastrointestinal malignancies, FGFR2 status was assessed using an additional dual-color break-apart fluorescence in situ hybridization (FISH) assay. No FGFR2 rearrangements were identified in any of the analyzed tumors. However, one case exhibited increased FGFR2 signal copy number without evidence of structural rearrangement.

Additional immunophenotypic analyses were performed to further investigate the biological relationship between the adenocarcinoma and yolk sac tumor (YST) components (Table 3). Claudin18.2 expression showed heterogeneous compartment-specific patterns across the three tumors. One case demonstrated strong membranous expression in both adenocarcinoma and YST components, one case showed moderate-to-strong expression in the adenocarcinoma component with only focal staining in YST areas, whereas a third case exhibited focal expression limited to the adenocarcinoma component and complete loss of staining in the YST component. In contrast, HER2 expression demonstrated heterogeneous, compartment-specific patterns. In one HER2-amplified tumor, HER2 expression was maintained in both the adenocarcinoma and the YST components. In another HER2-positive case, however, expression was restricted to the adenocarcinoma component and lost in the YST areas. PD-L1 expression was absent in both tumor compartments in all analyzed cases. Representative immunohistochemical findings for Claudin18.2 and HER2 are shown in Supplementary Figure S4. These findings collectively provide additional evidence of phenotypic divergence between the adenocarcinoma and YST components. Detailed compartment-specific immunophenotypic and molecular findings are summarized in Table 3.

At the same time, the persistence of selected molecular alterations across tumor compartments is compatible with a shared epithelial origin. Furthermore, identifying ERBB2 amplification in a subset of tumors underscores the importance of comprehensive molecular and immunophenotypic characterization in this rare gastric neoplasm.

## 3. Discussion

Primary gastric adenocarcinomas with yolk sac tumor (YST) differentiation represent an exceptionally rare and biologically intriguing phenotype, characterized by composite morphology and frequently aggressive clinical behavior [3,4,5,7,21,22]. The coexistence of glandular and extraembryonic elements, together with previously demonstrated molecular clonality, supports a model of somatic lineage reprogramming rather than aberrant germ cell migration [2,6,8]. In our series, the immunophenotypic profile, defined by diffuse SALL4, AFP, and Glypican-3 expression in the absence of OCT3/4 and PLAP, further supports primitive extraembryonic differentiation and aligns with emerging concepts of oncofetal reprogramming and tumor lineage plasticity in aggressive epithelial malignancies [15,16,17,18,19].

Genomically, these tumors exhibited microsatellite stability and intermediate tumor mutational burden, resembling conventional gastric adenocarcinoma more closely than canonical germ cell tumors [2,20]. Despite intertumoral heterogeneity at the gene level, partial convergence on proliferative and developmental signaling pathways was observed. A CCND3 missense alteration was identified in all three tumors. However, the biological significance of this finding remains uncertain and requires validation in larger cohorts. Additionally, alterations affecting receptor tyrosine kinase and PI3K signaling pathways suggest convergence on biologically relevant oncogenic networks despite substantial gene-level heterogeneity.

Collectively, these findings are compatible with the hypothesis that yolk sac differentiation may arise through epithelial-associated reprogramming; however, targeted sequencing alone cannot definitively exclude alternative germ cell-related mechanisms. Importantly, the observed retention of HER2 and/or Claudin18.2 expression in at least a subset of YST components further supports a shared epithelial lineage despite phenotypic divergence.

From a diagnostic perspective, these results underscore the importance of integrating morphologic, immunophenotypic, and molecular features to accurately classify gastric tumors with YST differentiation. In particular, distinction from poorly differentiated adenocarcinoma or metastatic germ cell tumors may be challenging based on morphology alone and requires a combined diagnostic approach incorporating oncofetal markers and genomic profiling. Interestingly, additional lineage-associated biomarkers demonstrated compartment-specific expression patterns. Given the recognized role of Claudin18.2 as a marker of gastric epithelial differentiation [27,28,29] and as a target of emerging therapeutic strategies in gastric cancer [30], its loss in YST areas may reflect progressive erosion of the gastric epithelial transcriptional program during lineage reprogramming [15,16,17,18].

While one HER2-amplified tumor maintained HER2 expression in both components, another demonstrated HER2 positivity exclusively within the adenocarcinoma component. These findings indicate that YST differentiation is not uniformly associated with complete loss of gastric lineage-associated markers. Together with the heterogeneous retention of HER2 expression, this supports a model of phenotypic remodeling rather than complete lineage replacement. Differential expression of gastric lineage and immune-related markers between adenocarcinoma and yolk sac tumor components further supports phenotypic divergence within a shared epithelial neoplasm. Within current WHO classification frameworks [31], this integrative strategy may help refine the characterization of this rare phenotype and distinguish it from other AFP-producing gastric carcinomas [9,13,14].

Integration of copy number profiling with orthogonal cytogenetic validation further refined the interpretation of structural alterations. High-level *ERBB2* amplification confirmed by FISH in a subset of tumors identifies HER2-driven signaling as a clinically relevant vulnerability, whereas *MDM2* alterations displayed heterogeneous patterns ranging from focal amplification to polysomy-associated gains. This distinction highlights the importance of combining sequencing-based approaches with cytogenetic validation to accurately interpret copy number events in rare tumor settings.

Importantly, the molecular landscape of gastric tumors with yolk sac differentiation only partially overlaps with classical germ cell tumor biology and instead recapitulates oncogenic circuitry typical of gastrointestinal carcinogenesis. The presence of *ERBB2* amplification, PI3K pathway perturbations, and cell-cycle dysregulation is compatible with an epithelial-associated oncogenic framework, consistent with somatic retro-differentiation and lineage plasticity [27].

From a translational standpoint, the identification of potentially actionable alterations, particularly ERBB2 amplification, supports the clinical utility of comprehensive genomic profiling in rare malignancies lacking standardized therapeutic approaches [23,24,25,26,27]. Although limited by the small cohort size, these findings suggest that treatment strategies may be more appropriately aligned with gastric adenocarcinoma paradigms rather than canonical germ cell tumor protocols, with HER2-directed therapies representing a potential option in selected cases [32].

Overall, our exploratory findings are compatible with the hypothesis that yolk sac differentiation may represent a phenotypic manifestation of epithelial tumor plasticity.

The observed compartment-specific expression of gastric lineage-associated markers further supports the hypothesis that yolk sac differentiation reflects phenotypic remodeling within an epithelial neoplasm rather than the emergence of an independent germ cell tumor. Taken together, the compartment-specific expression patterns that we observed for HER2 and Claudin18.2 suggest that yolk sac differentiation is associated with progressive phenotypic divergence rather than complete genomic separation. These observations are consistent with recent evidence demonstrating that somatically derived yolk sac tumors retain molecular features of their parental epithelial neoplasms while acquiring a yolk sac phenotype through lineage reprogramming [33].

This study has several limitations. The use of a targeted 523-gene panel without matched normal tissue limits comprehensive assessment of structural variation, mutational signatures, clonal architecture, and genome-wide evolutionary trajectories. Future studies integrating whole-exome or whole-genome sequencing, transcriptomics, and component-specific profiling will be necessary to clarify the molecular basis of yolk sac differentiation [34].

## 4. Materials and Methods

### 4.1. Targeted Next-Generation Sequencing (TruSight Oncology 500)

Targeted next-generation sequencing was performed using the TruSight Oncology 500 assay (Illumina), a hybrid-capture-based panel interrogating 523 cancer-related genes. The assay enables detection of single-nucleotide variants (SNVs), small insertions/deletions (indels), and copy number alterations, and provides estimates of tumor mutational burden (TMB) and microsatellite instability (MSI) status.

Formalin-fixed, paraffin-embedded (FFPE) tumor sections were reviewed by expert pathologists, and representative tumor areas with an estimated tumor cellularity > 50% were selected by expert pathologists. Genomic DNA was extracted using the AllPrep DNA FFPE Kit (Qiagen) according to the manufacturer’s instructions. Library preparation was performed following the TruSight Oncology 500 protocol, including hybrid-capture enrichment and bead-based normalization. Pooled libraries were sequenced on a NextSeq 500 platform (Illumina, San Diego, CA, USA). Primary data processing and variant calling were conducted using the TruSight Oncology 500 Local App (v1.3.1). Variant annotation and clinical interpretation were supported by the Clinical Genomic Workspace (PierianDx, San Diego, CA, USA).

### 4.2. Bioinformatic and Variant Interpretation Workflow

Bioinformatic analysis was performed using the Illumina TruSight Oncology 500 Docker pipeline (v2.1). Sequencing reads were aligned to the human reference genome GRCh38 (hg38). Variant annotation and clinical interpretation were conducted using complementary pipelines integrating the PierianDx Clinical Genomic Workspace and the Personal Cancer Genome Reporter (PCGR, v0.9.2). For database compatibility, clinical annotation within PCGR was mapped to GRCh37 (hg19). Within the PierianDx environment, filtering was restricted to non-synonymous coding variants with variant allele frequency > 5%, excluding alterations classified as benign or likely benign in curated clinical databases. In the absence of matched normal tissue, putative germline variants were minimized through population-frequency filtering and exclusion of variants classified as benign or likely benign in curated databases. Candidate variants were manually reviewed using the Integrative Genomics Viewer (IGV) to minimize FFPE-related artifacts, including deamination-associated transitions. Tumor mutational burden (TMB) was estimated using PCGR with calibration for targeted panel sequencing. Single base substitutions were classified according to the COSMIC pyrimidine-based convention, in which complementary substitutions are normalized to the pyrimidine context. Somatic copy number alterations were inferred based on fold-change thresholds, with high-level amplifications defined as fold-change > 2.0 (estimated copy number > 4). Variants were reported according to Human Genome Variation Society (HGVS) nomenclature and classified following AMP/ASCO/CAP guidelines. Pathway-level enrichment analyses were performed using GSEApy (v1.1.3) with Reactome, MSigDB Hallmark, and Gene Ontology reference sets. Data visualization was generated using GraphPad Prism (v9.0) and Python-based libraries (version 3.11).

### 4.3. Molecular Cytogenetics

Interphase fluorescence in situ hybridization (FISH) was performed on 3.5 µm formalin-fixed, paraffin-embedded (FFPE) sections assembled in tissue microarrays. *ERBB2* and *MDM2* copy number status was assessed using dual-color ZytoLight probe sets (ZytoVision GmbH, Bremerhaven, Germany). *ERBB2* was evaluated with the CEN17/SPEC *ERBB2* probe targeting 17q12 (*ERBB2*) and the D17Z1 centromeric control. *MDM2* was analyzed using the SPEC *MDM2*/CEN12 probe targeting 12q15 (MDM2) with D12Z3 as centromeric reference. FGFR2 rearrangements were assessed by FISH using a dual-color break-apart probe (ZytoLight SPEC FGFR2, ZytoVision, Bremerhaven-Fischereihafen, Germany) according to the manufacturer’s recommendations. Tumors were classified as rearranged when split signals were identified and as non-rearranged when a fused signal pattern was maintained.

Slides were processed using standardized pretreatment and hybridization conditions, including co-denaturation at 75 °C for 10 min and hybridization at 37 °C for 16 h, followed by post-hybridization stringency washes and nuclear counterstaining with DAPI. Signals were visualized using a fluorescence microscope (DM6000 Leica Biosystems, Deer Park, IL, USA) with appropriate filter sets, and enumeration was performed with CytoVision 7.7 software. For each case, at least 20 non-overlapping, morphologically intact interphase nuclei were independently scored by two observers. *ERBB2* amplification was defined according to current ASCO/CAP criteria for gastric carcinoma (*ERBB2*/CEN17 ratio ≥ 2.0). *MDM2* amplification was defined as an *MDM2*/CEN12 ratio ≥ 2.0 and/or the presence of large gene signal clusters (>10 signals per nucleus). Nuclei with weak hybridization signals, truncation artifacts, or excessive background autofluorescence were excluded from analysis.

## 5. Conclusions

We report the first detailed molecular characterization of gastric adenocarcinomas with yolk sac tumor (YST) differentiation, providing an exploratory integrated characterization of this rare phenotype and identifying molecular alterations that may be relevant for future studies. Integrated histopathologic, immunophenotypic, and genomic analyses are compatible with an epithelial origin with secondary extraembryonic reprogramming. The identification of alterations involving pathways such as ERBB2 and PI3K signaling underscores the clinical relevance of molecular profiling in this tumor subtype. Despite the limited cohort size, these findings provide a conceptual and translational framework to inform diagnosis and potential therapeutic strategies in rare gastric malignancies.

## Figures and Tables

**Table 1:** Clinicopathologic characteristics of gastric yolk sac (YST) cases.

Case	Age/Sex	Site	Surgery	Pathology	Stage: pTNM	Metastasis at Diagnosis	Outcome
1	68/M	Antrum	Exploratory laparotomy + peritoneal biopsies	YST + AC	pT3 pN1 R0 G3	LN, peritoneum	Died (11 months)
2	55/M	Antrum	Subtotal gastrectomy + D2 lymphadenectomy	YST + AC	pT1b pN1 R0 G2	LN	Alive (8 years)
3	75/M	Fundus/ stump	Total gastrectomy + lymphadenectomy	YST + AC	pT3 pN1 R0 G2	LN	Died (2 years)

AC: Adenocarcinoma; LN: Lymph node; YST: Yolk sac tumor.

**Table 2:** Selected Genomic Alterations with Potential Translational Relevance.

Gene	Alteration	Tier *	Clinical Relevance
**ERBB2**	Amplification	**IIC**	Established predictive biomarker in gastric adenocarcinoma
**PIK3CA**	Mutation	**IIE**	Potential pathway activation; investigational relevance
**FGFR4**	Gain/Mutation	**III**	Exploratory association; uncertain predictive significance
**KIT/PDGFRA**	Gain	**III**	Biological relevance unclear in this context
**MDM2**	Gain/Amplification	**IIE–III**	Potential investigational target

* Tier levels assigned according to the AMP/ASCO/CAP joint guidelines for somatic variant interpretation.

**Table 3:** Comparative immunophenotypic and molecular features of adenocarcinoma (ADC) and yolk sac tumor (YST) components.

Case	Component	HER2	Claudin18.2 (Intensity, % Positive Cells)	PD-L1	ERBB2 FISH	MDM2 FISH	FGFR2 FISH
**1**	ADC	3+	3+, (>75%)	−	Amplified	Amplified	Non-rearranged
	YST	2+	3+, (>75%)	−	Amplified	Amplified	Non-rearranged
**2**	ADC	3+	2+/3+, (55%)	−	Amplified	Polysomy 12	Non-rearranged (copy gain)
	YST	−	Focal, (<10%)	−	Negative	Polysomy 12	Non-rearranged (copy gain)
**3**	ADC	−	Focal, (<10%)	−	Negative	Negative	Non-rearranged
	YST	−	-	−	Negative	Negative	Non-rearranged

## Data Availability

The original contributions presented in this study are included in the article/Supplementary Materials. Further inquiries can be directed to the corresponding authors.

## Supplementary Materials

The following supporting information can be downloaded at: https://www.mdpi.com/article/10.3390/ijms27135786/s1.
