# Supplementary material for: Integrated Histopathologic and Targeted Genomic Characterization of Gastric Adenocarcinomas with Yolk Sac Tumor Differentiation
Source: Int J Mol Sci. 2026 Jun 26;27(13):5786. doi: 10.3390/ijms27135786 (PMC13362424; doi:10.3390/ijms27135786)
Supplement: Supplementary file 1 [file ijms-27-05786-s001.zip › Supplementary data.pdf]

Supplementary Table S1. Complete list of genomic alterations identified across the three gastric tumors with yolk sac differentiation. Sheet 1 reports single-nucleotide variants and small insertions/deletions (SNVs/indels), including gene symbol, genomic position, variant consequence, protein change, and variant allele frequency. Sheet 2 summarizes copy number alterations (CNVs), including chromosomal location, affected gene, estimated copy number, and copy number status.

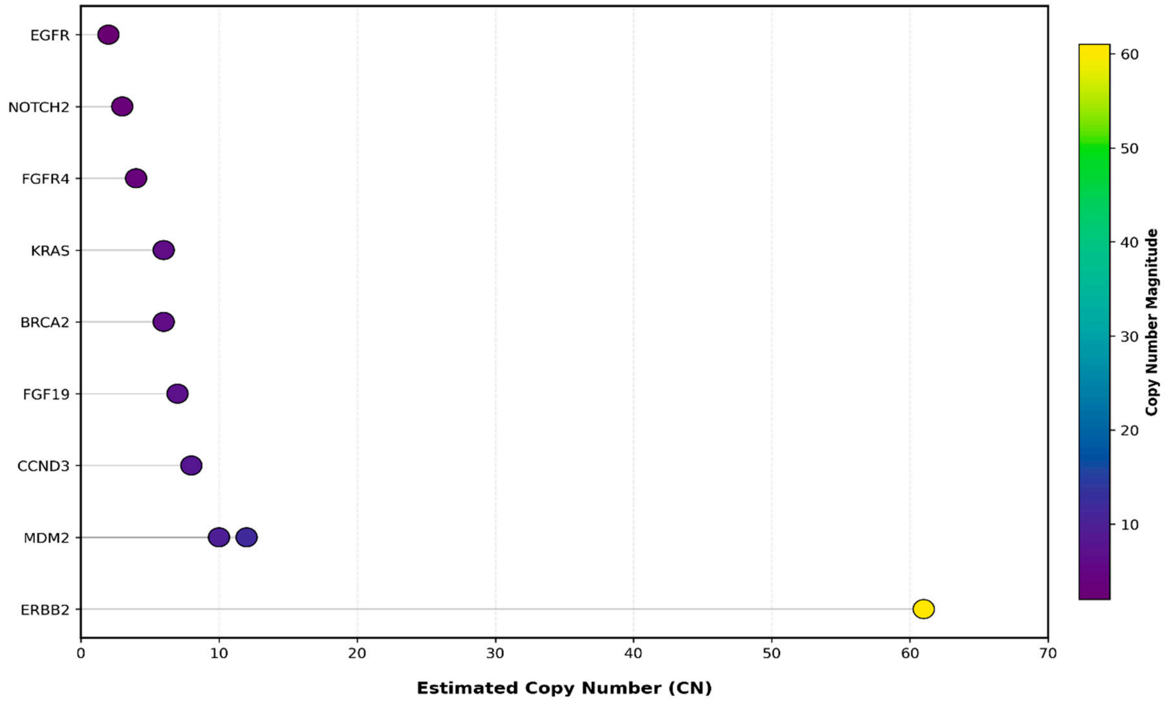

Supplementary Figure S1. Genome-wide distribution of copy number alterations across the three gastric tumors with yolk sac differentiation. Recurrent gains affecting oncogenic regulators, including *MDM2, CCND3*, and *ERBB2*, are highlighted.

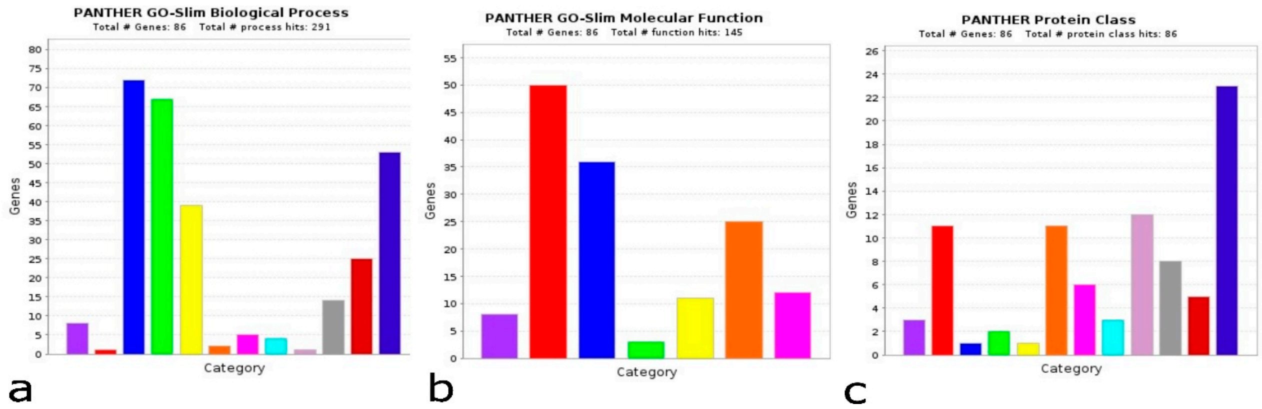

Supplementary Figure S2. Functional classification of altered genes using PANTHER and Gene Ontology annotation. Categories include biological processes and molecular functions enriched among genes affected by somatic alterations.

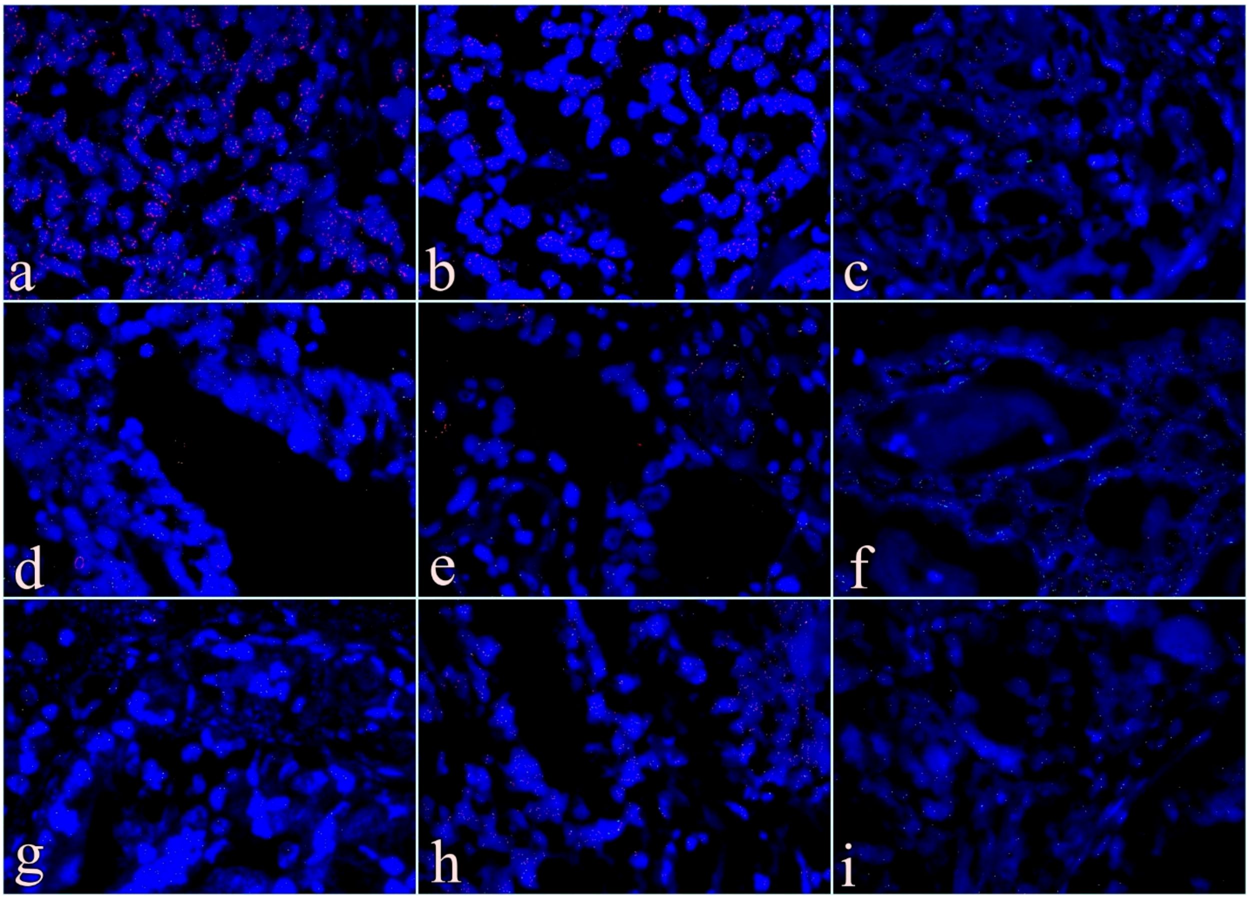

Supplementary Figure S3. **Representative FISH validation of ERBB2, MDM2 and FGFR2 alterations.** Panels (a-c) show ERBB2 status, including amplification in Cases 1 and 2 and a negative pattern in Case 3. Panels (d-f) illustrate MDM2 findings, including chromosome 12 polysomy, a negative case, and focal high-level amplification. Panels (g-i) depict FGFR2 break-apart FISH analysis, demonstrating the absence of FGFR2 rearrangements in all cases. Nuclei were counterstained with DAPI (blue).

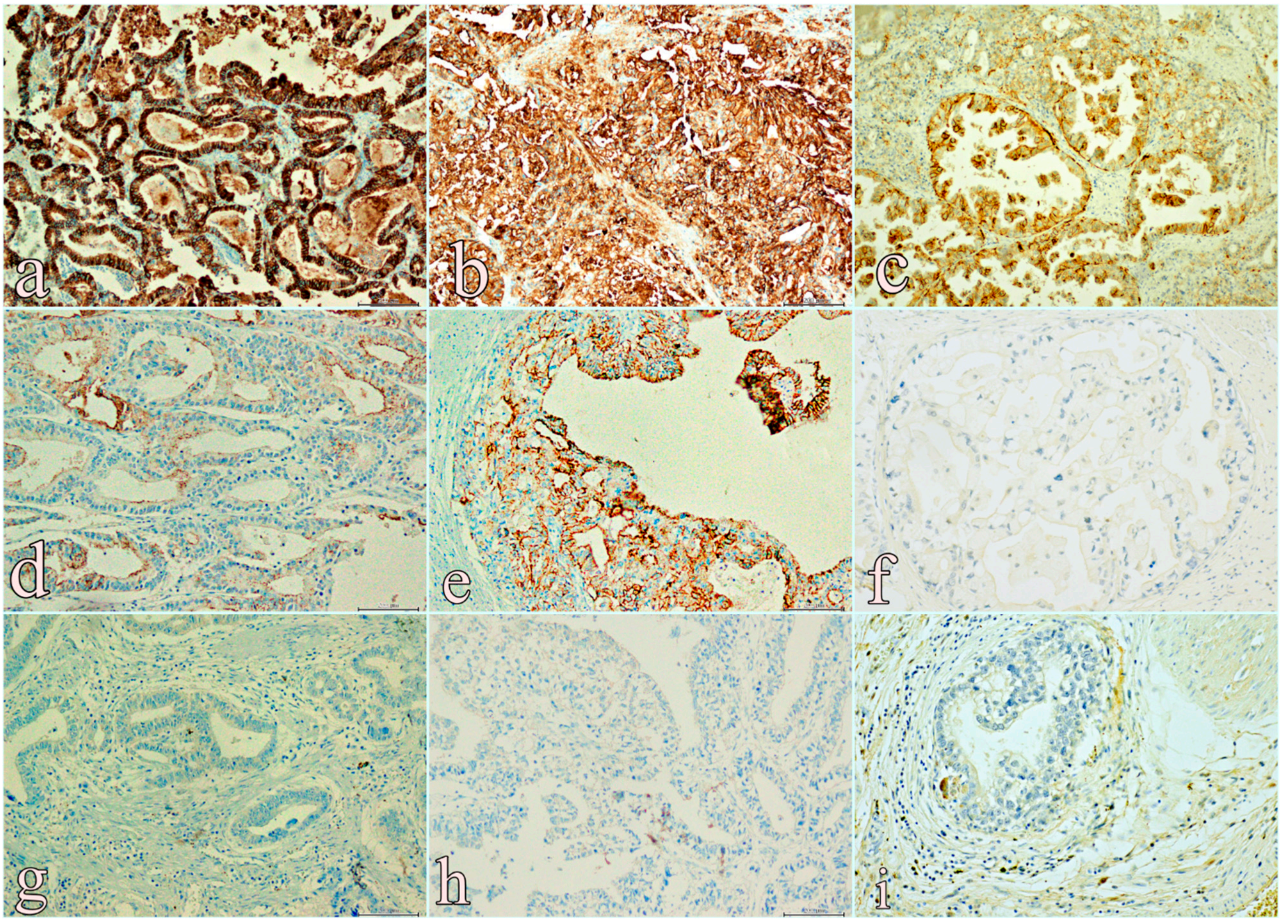

Supplementary Figure S4. **Representative Claudin18.2 and HER2 immunohistochemical expression in gastric adenocarcinomas with yolk sac tumor differentiation.** Panels a) -c), d)-f), and g)-i) correspond to Cases 1, 2, and 3, respectively. Claudin18.2 showed heterogeneous expression across adenocarcinoma and yolk sac tumor components, whereas HER2 expression was retained in the adenocarcinoma component of Cases 1 and 2. Original magnification ×200.
